# Insights into trehalose synthase function in the rhizobium *Paraburkholderia phymatum* STM815^T^ under abiotic stresses and during symbiosis

**DOI:** 10.1093/sumbio/qvag013

**Published:** 2026-04-03

**Authors:** Daphne Golaz, Gabriella Pessi

**Affiliations:** Department of Plant and Microbial Biology, University of Zurich, 8057 Zurich, Switzerland; Department of Plant and Microbial Biology, University of Zurich, 8057 Zurich, Switzerland

**Keywords:** compatible solute, nodule, sugar, cold stress, oxidative stress, filaments

## Abstract

The beta-proteobacterium *Paraburkholderia phymatum* STM815^T^ is a diazotrophic rhizobium that is highly competitive for legume root nodulation and is resistant to abiotic stresses, including heat, low pH, or drought stress. A gene coding for a potential trehalose synthase and localized on the symbiotic plasmid (_Sp_*treS*) was among the most highly upregulated genes in microoxic conditions and in symbiosis. In this study, we observed that cells grown in microaerobic conditions produced 5.8 times more trehalose than in aerobic conditions. Accordingly, a *P. phymatum*_Sp_*treS* mutant accumulated 29.5% less trehalose than the wild-type strain in microaerobic conditions. During symbiosis with common bean, the _Sp_*treS* mutant showed a 58.8% lower nitrogenase activity compared to the wild-type strain. Furthermore, in absence of _Sp_*treS P. phymatum* was more sensitive to H_2_O_2_-induced oxidative stress and to freezing conditions and formed filaments. These results expand our knowledge on the behavior of this beta-rhizobium under microoxic conditions and on the role of the enzyme trehalose synthase in rhizobial resistance to abiotic stresses and in symbiotic efficiency.

**Sustainability statement**

This study aligns strongly with the United Nations Sustainable Development Goals (SDG) 2: Zero Hunger. The rhizobium *Paraburkholderia phymatum* performs biological nitrogen fixation (BNF) in symbiosis with a broad range of legumes and exhibits resistance to both abiotic and biotic stresses. These characteristics make rhizobia a sustainable alternative to synthetic fertilizers for increasing the yield of protein-rich staple crops. Furthermore, BNF reduces the environmental footprint of agriculture, lowering pollution, and promoting a more balanced soil microbiome These benefits also contribute to SDG 3: Good health and well-being.

## Introduction

Rhizobia are beneficial bacteria and major contributors to the nitrogen cycle since they are capable of nodulating legumes (Herridge et al. 2008, Masson-Boivin et al. 2009), and reduce the highly abundant atmospheric nitrogen (N_2_) into a form that the plant can use (Gopalakrishnan et al. 2015, Robertson and Groffman 2015, Grzyb et al. 2021). The symbiosis begins with the exchange of molecular signals between the two partners, leading to the development of a symbiotic organ (nodule) on the plant root (Ledermann et al. 2021b, Yang et al. 2022, Ninzatti et al. 2025). Inside the nodule, rhizobia differentiate into bacteroids that fix nitrogen through the nitrogenase enzyme in exchange for C_4_-dicarboxylates provided by the plant (Ledermann et al. 2021b, Lindström and Mousavi 2020). Because the nitrogenase is sensitive to O_2_, the conversion of N_2_ into ammonium occurs in a microoxic environment (Lindström and Mousavi 2020). *Paraburkholderia phymatum* STM815^T^ is a beta-proteobacterium that has been isolated from *Mimosa* root nodules and described for the first time in 2001 (Moulin et al. 2001, Vandamme et al. 2002). The genome of *P. phymatum* consists of two chromosomes and two plasmids (pBPHY01 and pBPHY02), the latter of which contains most of the genes that are important for symbiosis with legumes (Moulin et al. 2014). *Paraburkholderia phymatum* is a promiscuous symbiont that can form a symbiosis with >50 members of the *Fabaceae* and *Mimosoideae* families and is highly competitive for root nodulation against other members of the soil microbiota (Elliott et al. 2007, Melkonian et al. 2014, Campos et al. 2017, Lardi et al. 2017a, Bellés-Sancho et al. 2021b, 2023). Furthermore, *P. phymatum* can also fix nitrogen in free-living conditions (Elliott et al. 2007), and is highly resistant to abiotic stresses like acidity, drought, salinity, space-mimicking conditions, or heavy metal-induced stresses (Mishra et al. 2012, Talbi et al. 2013, Liu et al. 2020, Golaz et al. 2024, Golaz et al. 2025).

*Paraburkholderia phymatum ' s* ability to tolerate environmental stresses while maintaining effective symbiosis highlights its potential as a bioinoculant. In fact, biological nitrogen fixation through rhizobial crop inoculation is an alternative to the systematic use of synthetic nitrogen fertilizers in farming (Fowler et al. 2013, Choudhary and Varma 2015, Abd-Alla et al. 2023). A previously conducted transcriptome analysis showed that several genes potentially involved in resistance to stress were highly upregulated in *P. phymatum* cells during symbiosis, or during growth in microoxic conditions that mimic the environment rhizobia encounter within nodules (Lardi et al. 2017b). Among the most upregulated genes in *P. phymatum* during symbiosis was Bphy_7407, which encodes a potential trehalose synthase TreS that catalyzes the reversible conversion of maltose to trehalose. This gene is localized on the symbiotic plasmid and is therefore referred to as _Sp_*treS*. Trehalose is a nonreducing disaccharide produced by a variety of organisms and serves as an energy or carbon storage and signaling molecule, but is best known for its important role as a compatible solute (Elbein et al. 2003, Schindler et al. 2021). A compatible solute is defined as a small organic molecule whose accumulation does not hinder normal cellular functions and protects the cell against a variety of stresses (Welsh 2000). The nonreducing disaccharide trehalose can mitigate the damage caused by exposure to oxygen radicals, heat, and desiccation in cells and cellular proteins (Benaroudj et al. 2001, Reina-Bueno et al. 2012, Martinet et al. 2019, Kuczyńska-Wiśnik et al. 2024). Indeed, trehalose accumulation prevents protein aggregation and acts as a molecular chaperone by inhibiting the unfolding of polypeptide chains (Benaroudj et al. 2001). Moreover, the disaccharide contributes to protein stabilization and membrane protection in bacteria (Leslie et al. 1995, Hincha and Hagemann 2004). Trehalose biosynthesis in *Rhizobium etli* and *Mesorhizobium ciceri* is important for survival to heat and osmotic stress in free-living conditions (Reina-Bueno et al. 2012, Moussaid et al. 2015) and, in *Bradyrhizobium diazoefficiens* USDA110, a trehalose biosynthesis mutant induced the formation of malformed and nonfixing soybean nodules.

Yet, the mechanisms linking trehalose production and symbiotic performances in rhizobia remain unclear (Streeter and Gomez 2006, Sugawara et al. 2010). As a compatible solute, trehalose has been reported to support bacterial survival under environmental stress, both in free-living conditions and during the early stages of the nodulation process (Sugawara et al. 2010, Ledermann et al. 2021a). Because stress reduces rhizobial competitiveness and nitrogen-fixation efficiency, trehalose may indirectly contribute to improved symbiotic performance (Sugawara et al. 2010, Ledermann et al. 2021a). Alternatively, trehalose and its derivatives, such as trehalose-6-phosphate, have been shown to play a role in signaling in plant–microbe interactions. Disruption of this signaling process could therefore reduce symbiotic efficiency (Paul 2007, Suárez et al. 2008, Domínguez-Ferreras et al. 2009).

Since little is known about the role of trehalose in *Paraburkholderia* species and its importance during symbiosis, we investigated the role trehalose synthase encoding gene located on *P. phymatum* symbiotic plasmid. Cells grown in oxygen-limited conditions accumulated more trehalose than those in aerobic conditions, and a *P. phymatum* mutant in _Sp_*treS* accumulated less trehalose than the wild type. Common bean nodules induced by the _Sp_*treS* mutant showed a 58.8% lower nitrogenase activity than those occupied by *P. phymatum* wild-type, suggesting a role of _Sp_*treS* in symbiosis. Finally, the _Sp_*treS* mutant was more sensitive to H_2_O_2_-induced oxidative stress and to freezing than the wild-type strain when grown under microoxic conditions and displayed longer filamentous cells.

## Materials and methods

### Bacterial strains and cultivation

Bacterial strains, plasmids, and primers used in this work are listed in Tables S1 and S2. Luria–Bertani (LB) rich medium was used to grow *E. coli* strains. *Paraburkholderia phymatum* strains were grown in modified LB prepared without salt (LB–NaCl) or in AB minimal medium (Clark and Maaløe 1967) with 15 mM succinate (ABS, Sigma-Aldrich, St. Louis, MO, USA) as a carbon source. For each biological replicate, 3 mL overnight cultures (in 20 mL glass tubes) obtained from a *P. phymatum* single colony were washed with 10 mM MgSO_4_ (Sigma-Aldrich, St. Louis, MO, USA) and reinoculated in oxygen-replete or limited conditions. For microoxic experiments, *P. phymatum* cultures were grown in 20 mL glass tubes filled with 14 mL of growth medium and incubated at 28°C and 180 rpm to reduce the oxygen-transfer coefficient. This setup follows the method originally developed by Cooper et al., and optimized by Paszti et al. (Cooper et al. 2003, Somerville and Proctor 2013, Paszti et al. 2025). Control samples grown in aerobic conditions were prepared in parallel in glass tubes filled with 3 mL of medium and incubated under the same environmental parameters.

### Plasmids and strains construction

*Paraburkholderia phymatum* STM815^T^ wild-type genomic DNA (gDNA) was isolated with a GenElute^TM^ Bacterial Genomic DNA kit (Sigma-Aldrich, St. Louis, MO, USA), and the plasmid DNA from *E. coli* strains was obtained using a QIAprep Spin Miniprep kit (Qiagen, Hilden, Germany). A Bphy_7407 (_Sp_*treS*) insertional mutant was constructed by using *P. phymatum* gDNA as a template to amplify a Bphy_7407 internal fragment (320 bp) with the primers Bphy_7407_F and Bphy_7407_R. The fragment was digested, inserted into the suicide vector pSHAFT-2 (pSHAFT-2::7407), and the resulting construct was used in a triparental mating with *P. phymatum* wild-type and the helper strain *E. coli* pRK2013. The mutant strain (_Sp_*treS* IM) was verified using the primers Bphy_7407_veri_F and Bphy_7407_veri_R. To construct the _Sp_*treS* IM complemented strain (_Sp_*treS* C), the _Sp_*treS* coding sequence with its native promoter was amplified using the primers Bphy_7407_c_F_nat_EcoRI and Bphy_7407_c_R_XbaI. The fragment was digested with the *Eco*RI and *Xba*I restriction enzymes and cloned into the pBBR1MCS2 plasmid (Kovach et al. 1995). The pBBR1MCS2:: Bphy_7407 plasmid was verified by sequencing (Microsynth AG, St Gallen, Switzerland), and conjugated into _Sp_*treS* IM by triparental mating (Lardi et al. 2017b).

### Trehalose quantification

Five overnight cultures obtained from five single colonies were washed and inoculated into 3 and 14 mL LB–NaCl. The cells were harvested and washed twice with 10 mM MgSO_4_. After centrifugation for 10 min at 10 000 rpm the cell pellet was resuspended in 1 mL of deionized sterile water and heated at 95°C for 15 min. The cells were centrifuged again for 10 min at 10 000 rpm, the pellet was discarded, and the lysate was used to measure the intracellular trehalose concentration through a trehalose assay kit (Megazyme, Bray, Ireland) according to the manufacturer’s instructions. The trehalase enzyme specifically converts trehalose into gluconate-6-phosphate, forming NADPH in the process. The newly formed NADPH can then be measured at an absorbance of 340 nm (Hayner et al. 2017).

### Plant growth conditions, inoculation, harvest, and quantification of nitrogen fixation activity

Common bean (*Phaseolus vulgaris* cv Negro jamapa) seeds were surface sterilized with absolute ethanol and 35% H_2_O_2_ as previously described (Lardi et al. 2017a, Bellés-Sancho et al. 2022). The seeds were incubated for 48 h on 1% agarose plates at 28°C to germinate. The germinated seeds were then planted in autoclaved yogurt pots filled with vermiculite (VTT-Group, Muttenz, Switzerland) and 160 mL of Jensen medium prepared without nitrogen (Bellés-Sancho et al. 2022). Four *P. phymatum* biological replicates were inoculated on five germinated seeds to a final OD_600_ of 0.025, for 20 plants per strain. The beans were grown for 21 days with a day/night cycle of 16 and 8 h. The experiment was performed with a light intensity of 160 micromoles per square meter (µM), a temperature of 22°C/25°C, and 60% humidity. After 21 days, the plants were harvested, and their roots were collected in glass tubes. Then, acetylene was injected and nitrogenase activity was quantified using a gas chromatograph as previously described (Bellés-Sancho et al. 2021a, ). The number of nodules and their dry weight were also assessed.

### Assessment of bacterial resistance to H_2_O_2_-induced stress

To test *P. phymatum* survival to H_2_O_2_-induced oxidative stress, five biological replicates per strain of cells grown microaerobically in minimal medium ABS were washed and homogenized to an OD_600_ of 0.5. The cells were incubated in the presence of 20 mM H_2_O_2_ (Sigma-Aldrich, St. Louis, MO, USA) for 20 min at 28°C under agitation. The number of colony-forming units (CFU) before and after treatment was determined on LB–NaCl agar plates.

### Testing rhizobial survival to cold temperature stress

To determine *P. phymatum*’s survival in freezing conditions, three biological replicates per strain were grown overnight in microaerobic cultures. The cells were washed in LB–NaCl, homogenized to a final OD_600_ of 0.5 and incubated at −20°C for 3 h. The survival rate was quantified on LB–NaCl plates by comparing the CFU before and after freezing.

### Microscopy

Five µL of three biological replicates of *P. phymatum* cells grown in 14 mL LB–NaCl for 17 h, 28°C and 180 rpm, were dropped on a 0.8% agarose pad deposited at the surface of a glass slide. Confocal microscopy was then performed using an Olympus BX51 fluorescence microscope (Olympus Corporation, Tokyo, Japan) and a microscope digital camera system DP50 (Olympus Corporation, Tokyo, Japan) at a magnification of ×1000.

### Statistical analysis

Student *t*-test and ANOVA with Tukey multiple comparisons test were performed to analyze the results of the phenotypical assays (trehalose concentration, cell length, resistance to H_2_O_2_, plant test) using the licensed version of BioRender (Toronto, ON, Canada).

## Results

### *Paraburkholderia phymatum* produces more trehalose when grown in oxygen-limited conditions

**Figure 1:**
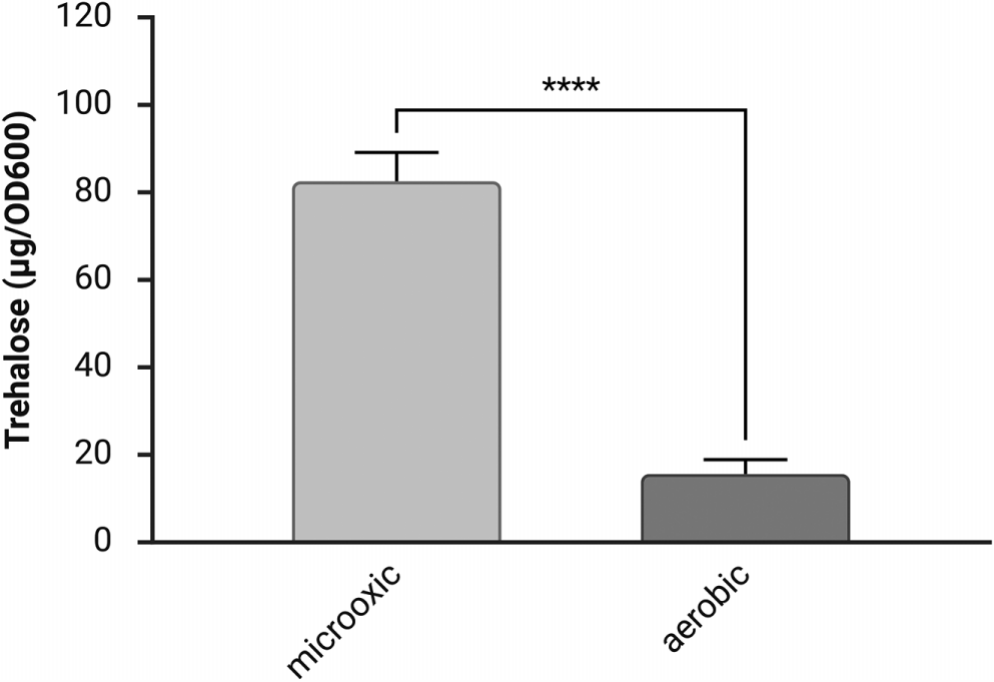
Intracellular trehalose concentration in *P. phymatum* wild-type cells grown in microaerobic and aerobic conditions. Cells incubated in LB–NaCl medium for 24 h in 20 mL tubes filled with 3 mL (aerobic) and 14 mL (microaerobic) were collected, and their lysate was used for trehalose quantification through an enzymatic assay. The standard deviations are given, and five (n = 5) biological replicates were performed. Differences between samples were analyzed with Student t-test. (*P-value < .05). Created with Biorender.com.

Previous studies showed that *P. phymatum* STM815^T^ Bphy_7407 was highly upregulated during symbiosis and microoxic growth conditions compared to growth in aerobic cultures (Lardi et al. 2017b). Bphy_7407 is located on the symbiotic plasmid and encodes a potential bidirectional trehalose synthase that we called _Sp_*treS* (Fig. S1). To assess if growth in oxygen-limiting conditions affects the accumulation of trehalose in *P. phymatum*, the intracellular concentration of the disaccharide was measured through an enzymatic assay able to distinguish between trehalose and maltose. For this, *P. phymatum* cells were grown in 20 mL glass tubes filled with 3 mL or 14 mL of LB–NaCl for 24 h, and the intracellular concentration of trehalose was measured. In line with the previous transcriptome analysis, *P. phymatum* wild-type cells grown in oxygen-limited conditions accumulated 5.8-fold more trehalose than those grown in aerobic conditions (Fig. 1). These results suggest that exposure to microoxia leads to trehalose accumulation in *P. phymatum*.

### Under microoxic conditions, a mutant in _Sp_*treS* produces less trehalose than the wild type

**Figure 2:**
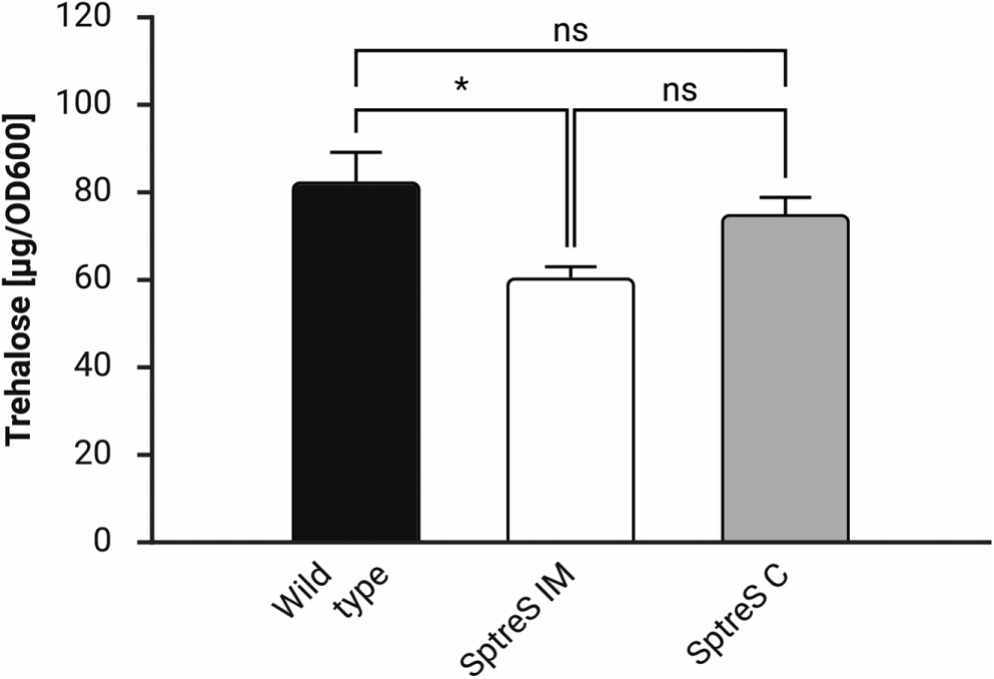
Intracellular trehalose concentration in *P. phymatum* wild-type (Wild-type), _Sp_*treS* mutant (SptreS IM) and _Sp_*treS* complemented strain (SptreS C) grown in oxygen-limited conditions. Cells incubated in glass tubes filled with 14 mL LB–NaCl medium for 24 h were spun down, lysed, and their trehalose concentration was assessed through an enzymatic assay. The standard deviations are given, and six (*n* = 6) biological replicates were performed. Differences between samples were analyzed with one-way ANOVA with Tukey multiple comparisons test (*P-value < .05, ns: nonsignificant). Created with Biorender.com.

To assess the role of _Sp_*treS* in trehalose synthesis, we constructed a *P. phymatum*_Sp_*treS* mutant (_Sp_*treS* IM) and a complemented strain (_Sp_*treS* C). Whereas there was no difference in growth rate between the wild type, _Sp_*treS* mutant and complemented strain in microoxic conditions (Fig. S2A and B), the _Sp_*treS* mutant accumulated 29.5% less trehalose than the wild-type strain and we could partially complement the phenotype by adding back _Sp_*treS* on a plasmid (Fig. 2). This result indicates that _Sp_*treS* contributes to the production of trehalose in *P. phymatum* grown in oxygen-limited conditions.

### The presence of *P. phymatum*_Sp_*treS* positively affects nitrogenase activity during symbiosis with common bean

**Figure 3:**
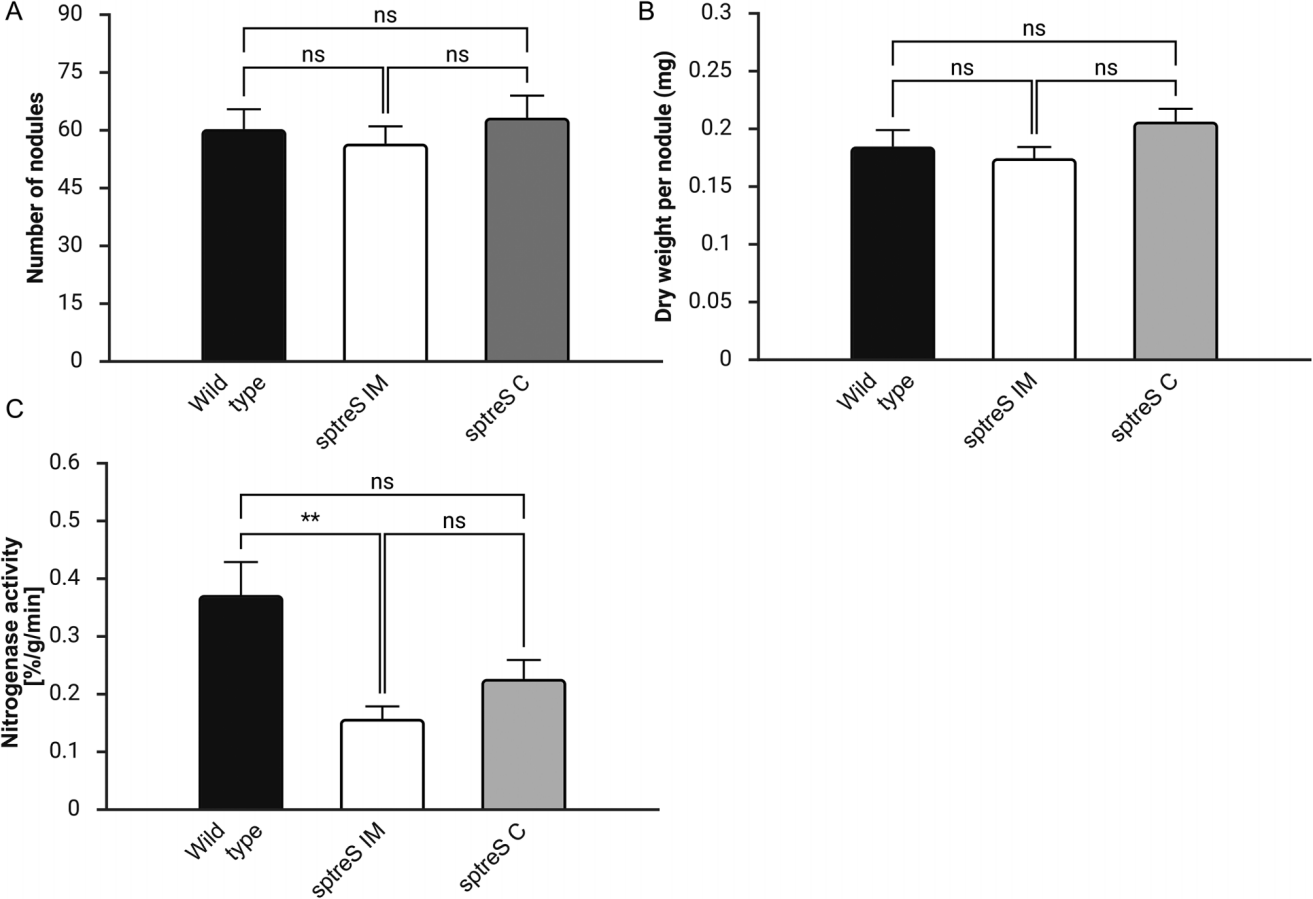
Symbiotic properties of *P. phymatum* wild-type (wild-type), _Sp_*treS* mutant (SptreS IM), and _Sp_*treS* complemented strain (SptreS C) in common bean. Number of nodules (A), nodules dry weight (B), and normalized nitrogenase activity (C) after 21 days of incubation in a greenhouse with a 16 h/8 h day/night cycle, an illumination of 160 µM, a temperature of 22°C/25°C, and 60% humidity. There were four (*n* = 4) bacterial biological replicates, to inoculate five plants each. Statistical analysis was performed through one-way ANOVA with Tukey multiple comparisons. Error bars indicate the standard error of the mean; **P-value < .01; ns: nonsignificant. Created with Biorender.com

Because of its (i) increase in expression inside root nodules compared to free-living conditions and (ii) genomic localization on the symbiotic plasmid, the role of *P. phymatum*_Sp_*treS* in symbiosis was investigated using common bean as a model legume. For this, germinated seeds were inoculated with *P. phymatum* wild-type, _Sp_*treS* mutant strain, or the complemented strain _Sp_*treS* C and incubated for 21 days in the greenhouse. While all the inoculated plants showed a similar number of nodules and dry weight (Fig. 3A and B), the nodules induced by the _Sp_*treS* mutant displayed 58.8% lower nitrogenase activity compared to nodules occupied by *P. phymatum* wild-type (Fig. 3C). Nitrogenase activity was partially complemented after the addition of a plasmid containing _Sp_*treS* into the _Sp_*treS* mutant. These results suggest that *P. phymatum* trehalose synthase encoded by _Sp_*treS* is dispensable for symbiosis establishment but is important for efficient nitrogenase activity.

### _Sp_*treS* is important for resistance to H_2_O_2_-induced oxidative stress

**Figure 4:**
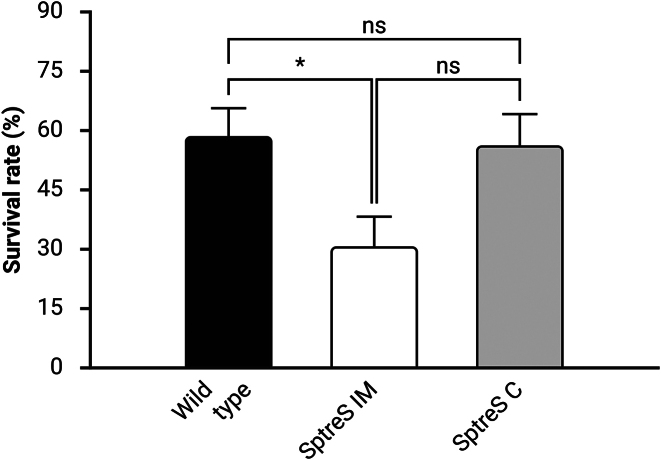
_Sp_treS-dependent survival rate of cells subjected to H_2_O_2_-mediated oxidative stress. CFU of *P. phymatum* wild-type (Wild type) and _Sp_*treS* mutant (SptreS IM) and complemented strain (SptreS C) grown in ABS liquid medium and treated with 20 mM for 20 min were counted and the survival rate was calculated according to the CFU after the treatment compared to before exposure to H_2_O_2_. Five (*n* = 5) biological replicates were tested. The standard errors are shown as bars, and a one-way ANOVA with Tukey multiple comparisons testwas performed; *P-value < .05; ns: nonsignificant. Created with Biorender.com.

Rhizobia have to overcome high levels of oxidative stress during the root infection process to establish a symbiosis with legumes (Reina-Bueno et al. 2012, Ledermann et al. 2021a). To assess the contribution of *P. phymatum*_Sp_*treS* to oxidative stress resistance, the survival of *P. phymatum* wild-type, the _Sp_*treS* mutant, and the complemented strain to H_2_O_2_ exposure was tested in microaerobic growth conditions. As shown in Fig. 4, _Sp_*treS* mutant cells were 47.7% more sensitive to 20 mM H_2_O_2_ than the wild type, and this defect is complemented by the addition of _Sp_*treS*. The fact that cells from the _Sp_*treS* mutant survived less than the wild type to H_2_O_2_ suggests that _Sp_*treS* plays a role in *P. phymatum*’s ability to resist oxidative stress.

### _Sp_*treS* mutant cells are more sensitive to cold stress than the wild-type strain

**Figure 5:**
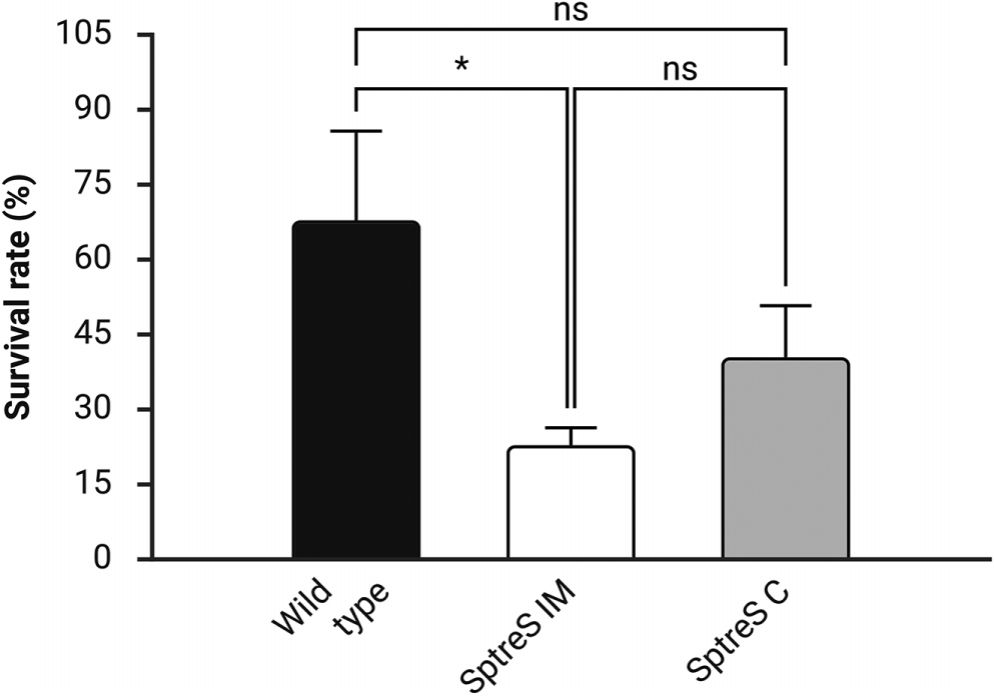
_Sp_*treS* affects *P. phymatum*’s survival at –20°C. The survival rate of *P. phymatum* wild-type, _Sp_*treS* IM, and _Sp_*treS* C was quantified by counting the CFU before and after cold treatment. Overnight cultures were washed in LB–NaCl, homogenized to an OD_600_ of 0.5, and incubated for 3 h at −20°C. Three (*n* = 3) biological replicates were assessed. The standard errors are shown as bars. An ANOVA with Tukey multiple comparisons test was conducted; *P-value < .05; ns: nonsignificant. Created with Biorender.com.

To follow our own observation that in the absence of _Sp_*treS, P. phymatum* cells survive less to storage at −80°C in the presence of 25% glycerol, the _Sp_*treS*-dependent ability of *P. phymatum*’s to withstand exposure to −20°C was assessed. For this, the number of *P. phymatum* wild-type, _Sp_*treS* IM, and _Sp_*treS* C cells grown microaerobically was determined before and after the strains were incubated at −20°C for 3 h. While 67.8% of *P. phymatum* wild-type cells survived to cold exposure, only 22.8% of the _Sp_*treS* mutant cells survived (Fig. 5). This phenotype was only partially restored to wild-type levels by adding _Sp_*treS* in trans on a plasmid.

### _Sp_*treS* mutant cells are longer and tend to form filaments

**Figure 6:**
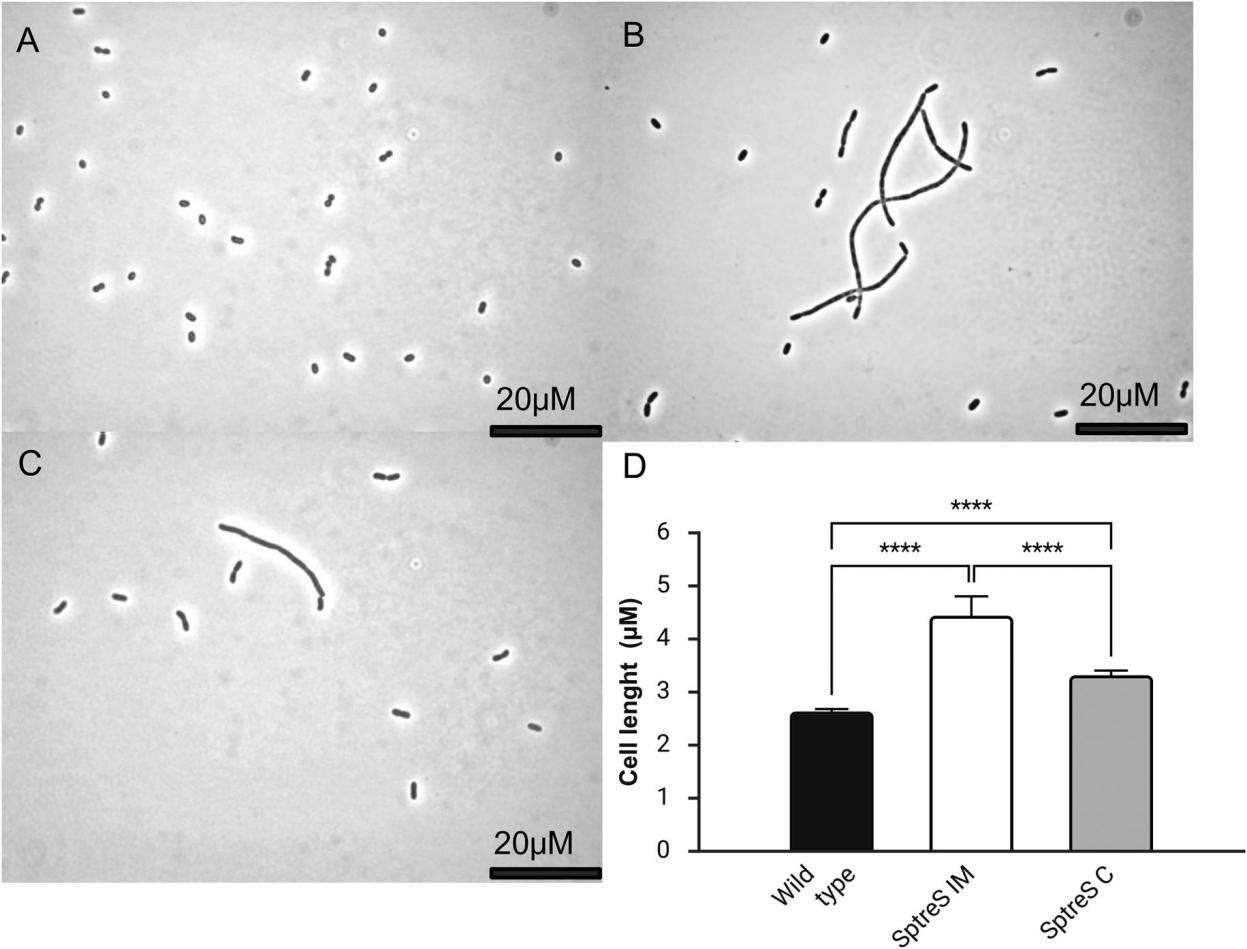
Mutation in _Sp_*treS* affects cell morphology and length. Representative confocal microscopy pictures of *P. phymatum* wild-type (A), _Sp_*treS* IM (B), and _Sp_*treS* C (C). The cells were grown in LB−NaCl rich medium at 28°C and 180 rpm for 17 h. (D) There were three (*n* = 3) biological replicates. A minimum of 200 cells per strain were measured from pictures, and the standard deviations are indicated as bars. Statistical analysis was conducted through an ANOVA with Tukey multiple comparisons test; ***P-value < .001. Created with Biorender.com.

To test if the presence of trehalose has an effect on cell morphology, confocal microscopy was performed. For this, *P. phymatum* wild-type, _Sp_*treS* IM, and _Sp_*treS* C cells were grown microaerobically in LB−NaCl rich medium to an OD_600_ of 0.5 (exponential phase) and prepared for microscopy as described in the Materials and Methods section. While *P. phymatum* wild-type cells displayed a regular rod-shaped morphology (Fig. 6A), the _Sp_*treS* mutant tended to form longer, filamentous cells (Fig. 6B). Indeed, *P. phymatum* wild-type cells were on average shorter than the _Sp_*treS* mutant cells, with a length of 2.6 ± 0.6 µm and 4.4 ± 0.4 µm, respectively (Fig. 6D), and _Sp_*treS* C cells had an intermediary length (3.3 ± 0.1 µm) (Fig. 6C and D).

## Discussion

In this study, we investigated the role of a trehalose synthase TreS in the beta-rhizobium *P. phymatum*. This gene is localized on *P. phymatum* symbiotic plasmid (_Sp_*treS*) and showed high expression levels in symbiosis and in microoxic growing conditions (Lardi et al. 2017b). Moreover, _Sp_*treS* was among the most upregulated genes in *P. phymatum* cells grown under stressful space-mimicking conditions (Golaz et al. 2024). Rhizobia need mechanisms to withstand the various stresses they encounter in the soil and during the symbiotic process, and trehalose in alpha-rhizobia has been shown to play a role during the highly competitive early legume infection stage (Reina-Bueno et al. 2012, Ledermann et al. 2021a).

Here, we show that *P. phymatum* grown in oxygen-limited conditions accumulates significantly more intracellular trehalose than those incubated in aerobic cultures (Fig. 1), confirming the previous transcriptomic analysis that showed an increased expression of _Sp_*treS* (Bphy_7407) in microoxia (Lardi et al. 2017b). The trehalose synthase TreS is a bidirectional enzyme that converts maltose into trehalose (Sugawara et al. 2010, Ruhal et al. 2013, Lee et al. 2023). However, TreS has been shown to bind to maltose with a higher affinity than trehalose, making it the preferred substrate (Pan et al. 2004, Ruhal et al. 2013). A deeper look into *P. phymatum* genome revealed that the beta-rhizobium possesses two additional trehalose biosynthetic pathways. The first pathway for trehalose production consists of OtsA, a trehalose-6-phosphate synthase that metabolizes UDP glucose and glucose-6-phosphate into trehalose-6-phosphate (T6P), and OtsB, a T6P phosphatase that converts T6P into trehalose (Sugawara et al. 2010, Ruhal et al. 2013, Lee et al. 2023). Another pathway relies on TreY, a maltooligosyltrehalose synthase that converts maltooligosaccharides into maltooligosyl trehalose, and TreZ, a maltooligosyltrehalose trehalohydrolase, which transform it into trehalose (Sugawara et al. 2010, Ruhal et al. 2013, Lee et al. 2023). In our previous experiments, we observed that these genes were upregulated in cells exposed to a microaerobic environment, but not to the same high levels as _Sp_*treS* (Lardi et al. 2017b). Therefore, accumulation of trehalose in *P. phymatum* cells grown in microoxic conditions could be a defense mechanism against stress induced by exposure to an oxygen-limited environment. We show here that a *P. phymatum*_Sp_*treS* mutant grown under microoxic conditions has a lower trehalose content than the wild type (Fig. 2), indicating that the trehalose synthase is contributing to the accumulation of the disaccharide when exposed to low oxygen conditions (Cooper et al. , Paszti et al. ). However, the fact that the _Sp_*treS* mutant strain still produces trehalose suggests that the other trehalose synthesis pathways that are active in a _Sp_*treS* mutant, might be compensating for its function. Interestingly when the cells were grown in aerobic conditions, the _Sp_*treS* mutant had an intracellular trehalose content similar to that of *P. phymatum* wild-type (Supplementary Fig. S1), suggesting that _Sp_*treS* is mostly expressed in an oxygen-limited environment. A deeper look into the promoter region of the _Sp_*treS* gene revealed the presence of a potential binding site for the fumarate and nitrate reduction-like (Fnr) transcription factor. Interestingly, Fnr transcription factors are a family of regulators that activate the expression of genes involved in anaerobic and microoxic functions in bacteria (Mesa et al. 2006). In rhizobia, the Fnr-type regulator FixK_2_ controls the expression of genes essential for microoxic respiration in symbiosis (*fixNOQP* and *fixGHIS*) and nitrogen fixation (Mesa et al. 2006, Li and Li 2023). The enzyme nitrogenase, which converts N_2_ into ammonium, is sensitive to oxygen and requires a microoxic environment (roughly 11 nM of free oxygen) to function properly (Berger et al. 2018, Ledermann et al. 2021b). In line with this finding, the nodules induced by the _Sp_*treS* mutant showed a lower nitrogenase activity compared to the ones occupied by *P. phymatum* wild-type (Fig. 3). These observations suggest that trehalose is an important disaccharide for an efficient symbiosis with the beta-rhizobium *P. phymatum*. In fact, previous metabolomics experiments in other rhizobium-legume models showed that the precursor of trehalose (trehalose phosphate) is one of the metabolites that highly accumulate in nodules (Lardi et al. 2016). A 12-fold higher trehalose concentration was also observed in soybean *B. diazoefficiens* USDA110 bacteroids compared to cultivated cells, along with a 2.6-fold higher activity of the trehalose synthase enzyme (Streeter and Gomez 2006, Vauclare et al. 2013). Moreover, the number of *B. diazoefficiens* viable cells that were recovered from senescent soybean nodules has been shown to correlate with an abundance of trehalose, suggesting that the compatible solute is instrumental for enabling the long-term survival of bacteroids during symbiosis (Müller et al. 2001, Streeter 2003). Trehalose supplementation was shown to have a major impact on alpha-rhizobial survival to abiotic stresses such as drought. For instance, *B. diazoefficiens*’s survival to desiccation was improved by 294% (Streeter 2003, Sugawara et al. 2010), and a transcriptomic study conducted in *B. diazoefficiens* cells exposed to desiccation-induced stress showed an upregulation in *ots* and *treS* expression, both involved in trehalose synthesis (Cytryn et al. 2007). By mutating _Sp_*treS* we show here for the first time the role of a beta-rhizobial trehalose synthase in resistance to abiotic stress. The higher sensitivity of *P. phymatum* lacking _Sp_*treS* to H_2_O_2_-induced oxidative stress suggests that accumulation of the disaccharide protects the beta-rhizobium against oxygen radicals in microoxic environments (Fig. 4). This protective effect was not observed when *P. phymatum* was grown in aerobic conditions (Supplementary Fig. S4). This result implies that _Sp_treS is primarily advantageous under microoxic conditions, while other trehalose biosynthesis pathways may contribute to *P. phymatum* resilience in oxygen-repleted environments. Furthermore, the phenotype of the *treS* mutant was complemented by exogenous trehalose supplementation (Supplementary Fig. S5), confirming the role of trehalose in resistance to oxidative stress. The lower survival rate of the _Sp_*treS* mutant when subjected to freezing conditions compared to *P. phymatum* wild-type suggests that trehalose is also advantageous for surviving at low temperatures (Fig. 5). This observation is in agreement with another study conducted by Kandror and colleagues, which showed that trehalose production is essential for survival of *Escherichia coli* to cold stress (Kandror et al. 2002). Additionally, the extremophile *Glaciimonas* sp. PAMC28666 accumulates 13 times more trehalose when grown at 8°C than at 15°C, highlighting the importance of this compatible solute in cold tolerance (Shrestha et al. 2023). Future transcriptomic experiments aiming at studying how *P. phymatum* tolerates cold and freezing conditions might shed light on the regulatory mechanisms underlying adaptation to low temperatures. In fact, strains from the *Paraburkholderia* and *Burkholderia* genus are, in general, very versatile and have been shown to survive cold temperatures (Fernandez et al. 2012, Zakharova et al. 2021). *Burkholderia pseudomallei*, for instance, can withstand a temperature as low as −18°C and up to five successive thawing and refreezing steps (Zakharova et al. 2021). Another example is the endophyte *Paraburkholderia phytofirmans* PsjN, which helps to reduce chilling-induced damage in various plants such as grapevine, tomato, or *Arabidopsis thaliana* (Fernandez et al. 2012, Su et al. 2015, Licciardello et al. 2024). Future experiments conducted in legumes inoculated with *P. phymatum* wild-type, the trehalose mutant and a complemented strain and incubated in a freezing environment might reveal the role of _Sp_*treS* in protecting the plant against freeze damage. However, _Sp_*treS* seems not to play a role in resistance to osmotic stress induced by sorbitol (Supplementary Fig. S6), indicating that the trehalose synthase encoded on the symbiotic plasmid plays a major role in mitigating oxygen-related or cold-related stresses but not osmotic stress.

Another hint that _Sp_*treS* is important for resistance to abiotic stress in *P. phymatum* is the filamentous phenotype occurring in the absence of _Sp_*treS* and the restoration to the normal cell size after complementation of the mutant (Fig. 6). Cells are considered filamentous if they undergo a morphology switch that results in a length longer than five µM compared to their standard size (Fredborg et al. 2015). Certain bacteria switch to a filamentous morphology as a survival strategy to cope with stress, such as nutrient starvation or osmotic stress, and prevent cell division and septation during cell growth (Rizzo et al. 2020, Karasz et al. 2022). Yet nothing is known about the molecular mechanisms inducing filamentation in *P. phymatum*, and whether filamentation impacts other fitness traits, such as resistance to abiotic stresses and symbiosis. Prospective microscopic experiments conducted in cells grown under stress might clarify the advantages of switching to a filamentous morphology in *P. phymatum*.

Future experiments assessing the symbiotic performance of *P. phymatum* in legumes—either through exogenous trehalose addition or using a trehalose overproducing strain- might clarify how this disaccharide influences N_2_-fixing efficiency. In addition, an in-legume competition experiment with *P. phymatum* wild-type and a _Sp_*treS* mutant strain might help determine the role of trehalose in the competitive early step of root colonization. Ultimately, this work provides new information on changes that occur in the beta-rhizobium *P. phymatum* exposed to abiotic stresses and reinforces the potential of using this strain as a bioinoculant to improve plant growth in environmentally harsh conditions.

## Supplementary Material

qvag013_Supplemental_File

## Data Availability

All data are available in the main text, the Supplementary material and materials.
