# Supplementary material for: Insights into trehalose synthase function in the rhizobium Paraburkholderia phymatum STM815T under abiotic stresses and during symbiosis
Source: Sustain Microbiol. 2026 Apr 3;3(2):qvag013. doi: 10.1093/sumbio/qvag013 (PMC13375364; doi:10.1093/sumbio/qvag013)
Supplement: Supplementary file 1 [file qvag013_Supplemental_File.pdf]

Insights into trehalose synthase function in the rhizobium *Paraburkholderia phymatum* STM815^T under abiotic stresses and during symbiosis

Daphne Golaz, Gabriella Pessi

Department of Plant and Microbial Biology, University of Zurich, Zurich, Switzerland

Correspondence: gabriella.pessi@botinst.uzh.ch

Supplementary information

Supplementary Figures S1 to S2

Supplementary Tables S1 to S6

Supplementary References

Supplementary figures

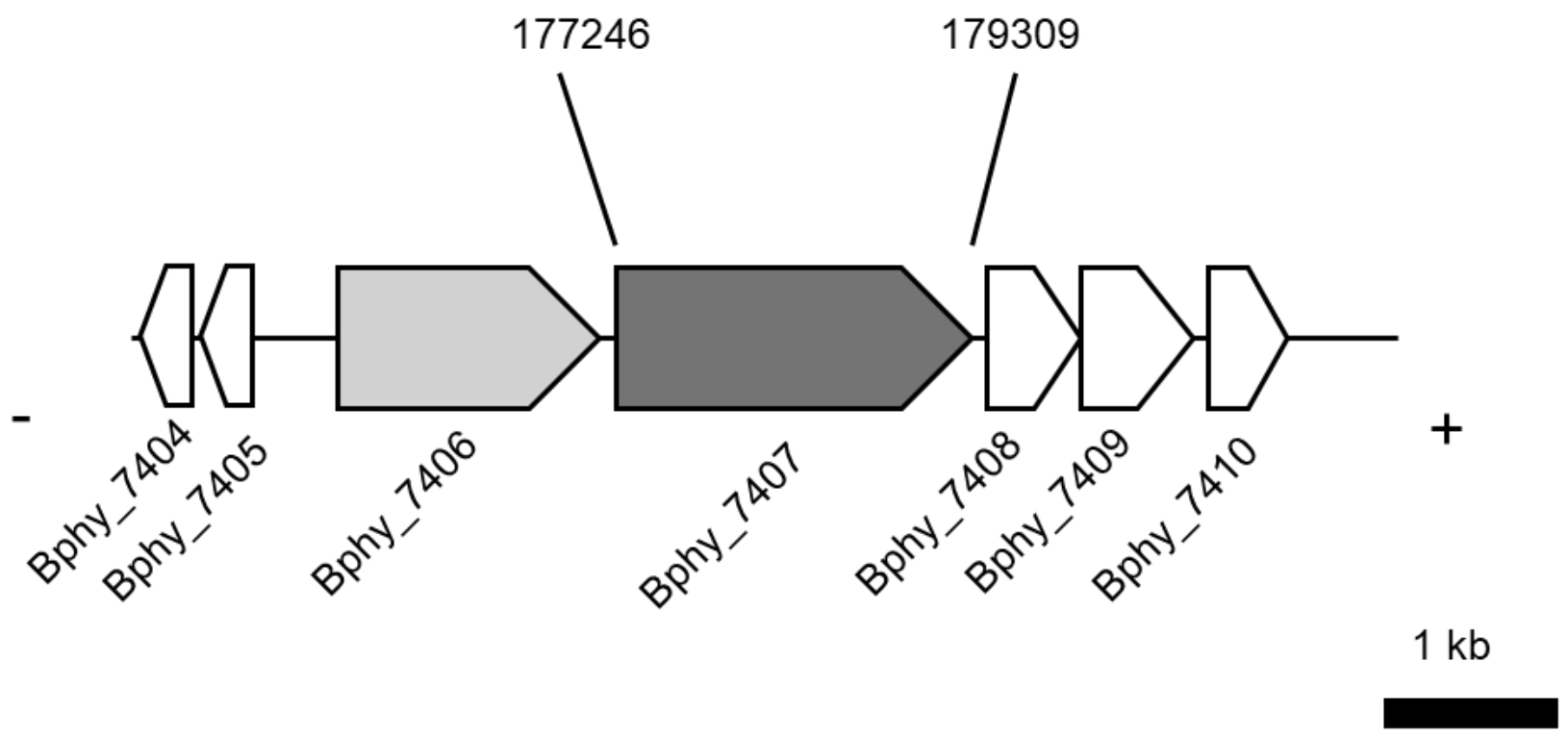

Bphy_7404: unknown protein
Bphy_7405: unknown protein
Bphy_7406: aldehyde dehydrogenase
Bphy_7407: trehalose synthase SptreS
Bphy_7408: unknown protein
Bphy_7409: unknown protein
Bphy_7410: unknown protein

Figure S1: Genomic localization of Bphy_7407. The numbers on top refer to the nucleotides position in the genome according to the Burkholderia database (https://www.burkholderia.com). Genes encoding proteins whose functions are not predicted are in white.

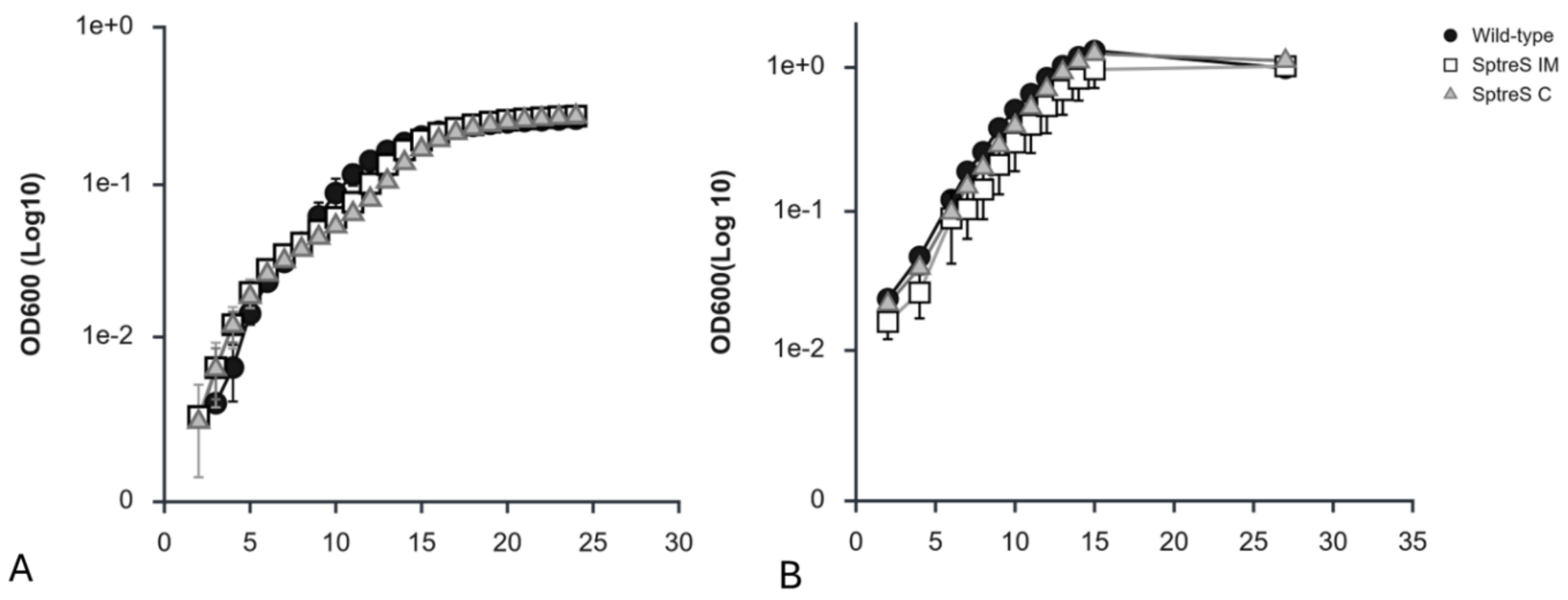

Figure S2: Growth profile of *P. phymatum* wild-type (Wild-type), a ${}_{Sp}$*treS* mutant (SptreS IM) and the complemented strain of ${}_{Sp}$*treS* (SptreS C). The cells were grown in 100 µL of ABS minimal media, at 28°C to measure growth in microaerobic conditions (**A**). The strains were inoculated in 25 mL ABS minimal medium in 100 mL flasks and incubated at 28 °C, 180 rpm to assess growth in aerobic conditions (**B**). No difference in growth was observed between the strains in either oxygen level. Three (n=3) biological replicates were performed. The standard deviations are indicated as bars. Created with Biorender.com

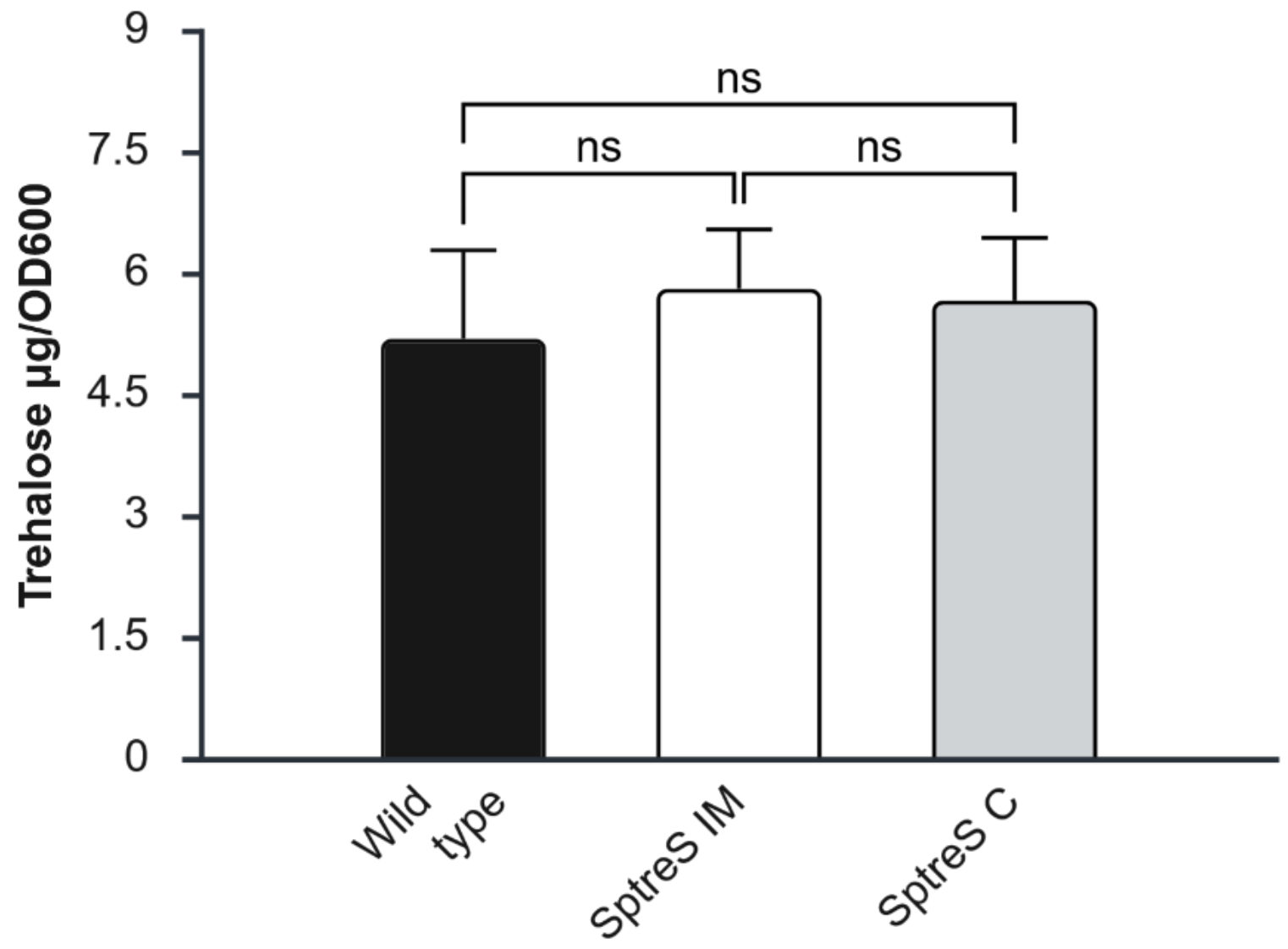

Figure S3: Intracellular trehalose concentration in *P. phymatum* wild-type (Wild type), $_{Sp}$*treS* mutant (SptreS IM) and $_{Sp}$*treS* complemented strain (SptreS C) grown in oxygen-replete conditions. Cells incubated in 3 mL LB-NaCl medium for 24 hours were precipitated and lysed. The intracellular trehalose concentration was quantified through an enzymatic assay. Six (n=6) biological replicates were tested, and the standard deviations are indicated as bars. ns: non-significant. Statistical analysis was performed through one-way ANOVA with Tukey multiple comparisons test. Analyzed with Biorender.com.

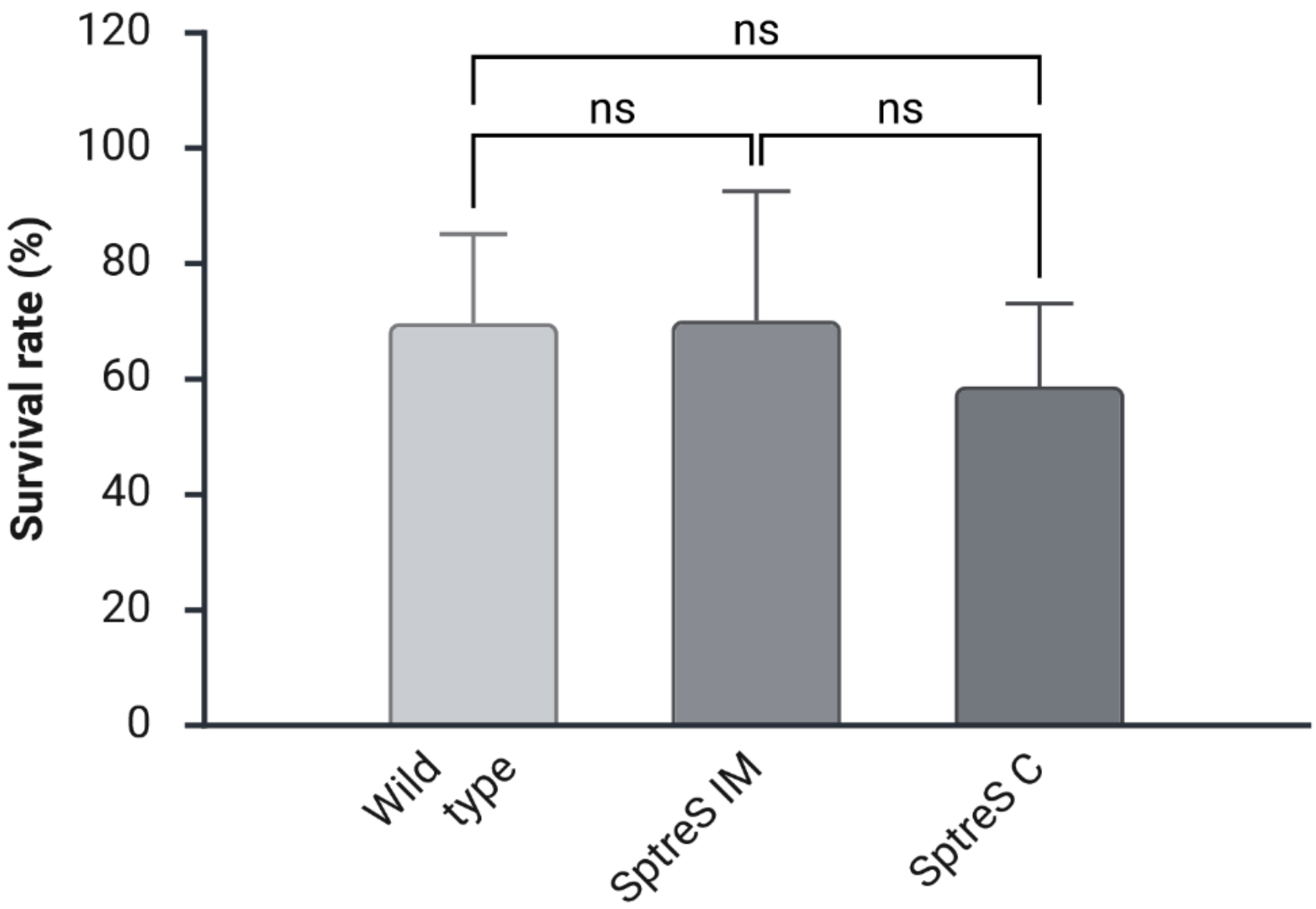

Figure S4: $_{Sp}$treS-dependent resistance to H_2O_2-induced oxidative stress in *P. phymatum* cells grown in oxygen-replete conditions. Colony-forming units from *P. phymatum* wild-type (Wild type), SptreS mutant (SptreS IM), and $_{Sp}$*treS* complemented strain (SptreS C) from aerobic ABS minimal medium overnight cultures were counted before and after treatment with 20 mM H_2O_2 to assess the survival rate. Three (n=3) biological replicates were performed; ns: non-significant. The standard deviations are shown as bars. The statistical analysis was conducted through a one-way ANOVA with Tukey multiple comparisons test. Analyzed with Biorender.com.

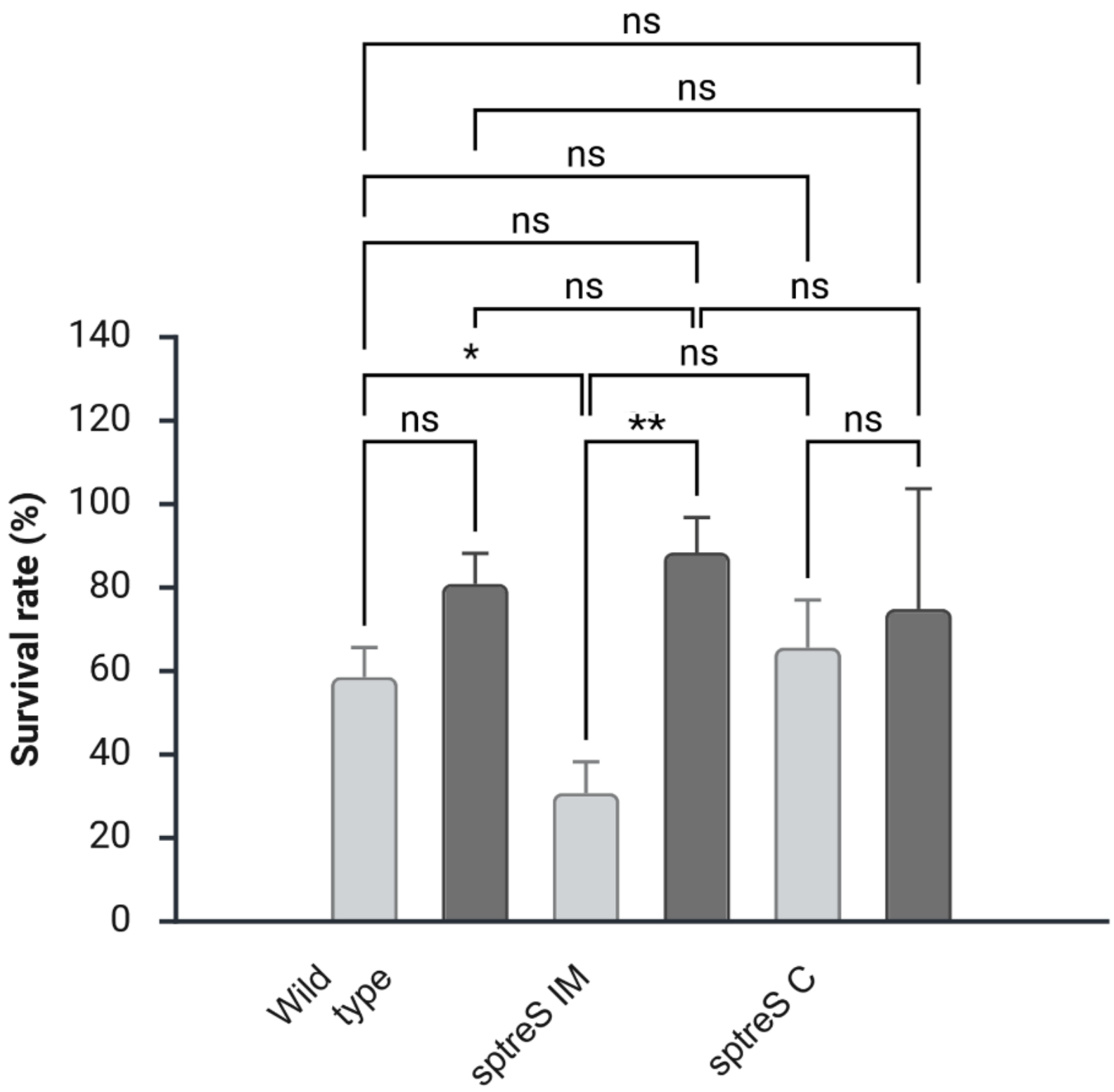

Figure S5: *P. phymatum* wild-type (Wild type), $_{Sp}$*treS* mutant (*SptreS* IM), and $_{Sp}$*treS* complemented (SptreS C) resistance to H_2O_2-induced oxidative stress before (light grey bars) and after treatment with 20 mM of trehalose (dark grey bars). One mL of the ABS overnight microaerobic culture was collected, and the remainder of the culture was supplemented with trehalose to a final concentration of 20 mM and incubated for 1 hour at 28°C and 180 rpm. The cells were washed, homogenized to a final OD_{600} of 0.5, and treated with 20 mM H_2O_2 for 20 minutes. The survival rate was calculated using CFU before and after H_2O_2 treatment. The standard deviations are indicated as bars. *: *p*-value < 0.05; **: *p*-value < 0.01; ns: non-significant. Three (n=3) biological replicates were performed. Statistical analysis was performed through one-way ANOVA with Dunn's comparison. Analyzed with Biorender.com.

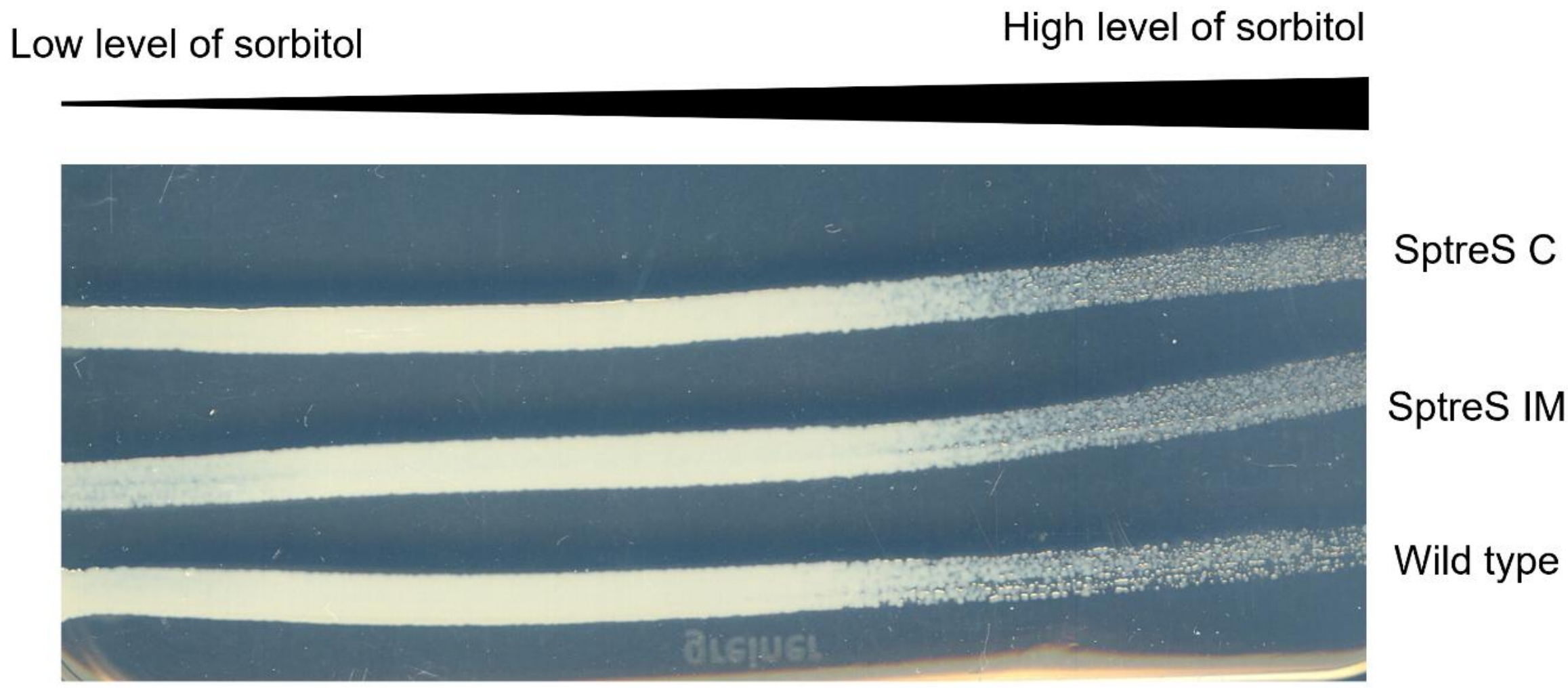

Figure S6: Resistance to osmotic stress in *P. phymatum* wild-type (Wild type), $_{Sp}$*treS* mutant (SptreS IM) and $_{Sp}$*treS* complemented strain (SptreS C). Growth was tested on linear gradient plates prepared with ABS minimal medium prepared with 400 mM sorbitol for the bottom layer. ABS was used for the upper layer. The cells were incubated for 96 hours at 28°C.

Supplementary tables

Table S1: Strains and plasmids used in this study

Strain or plasmid	Description	Antibiotics	Reference
E. coli			
Top10	*ΔlacX74 araΔ139Δ (ara-leu*)	none	Invitrogen
P. phymatum			
STM815^T	Wild-type	None	(Moulin et al. 2001)
$_{sp}$*treS* IM	Insertion mutant for the sp*treS* gene (Bphy_7407)	Chloramphenicol	This study
$_{sp}$*treS* C	Insertion mutant for the sptreS gene (Bphy_7407) complemented with pBBR1MCS2::Bphy_7407	Chloramphenicol, kanamycin	This study
**Plasmids**			
pRK2013	Helper plasmid	Kanamycin	(Figurski & Helinski,1979)
pSHAFT2	Suicide plasmid	Chloramphenicol	(Shastri et al. 2017)
pSHAFT2-sp*treS*	Suicide plasmid containing 320 bps internal fragment of Bphy_7407 for mutagenesis	Chloramphenicol	This study

pBBR1MCS2	Broad host range cloning vector	Kanamycin	(Kovach et al. 1995)
pBBR1MCS2 ::Bphy_7407	pBBR1MCS2 containing Bphy_7407 gene	Kanamycin	This study

Table S2: List of primers used in this study

Oligonucleotide	Sequence	Reference
Bphy_7407_F	GCGCgaattcAACTTTGATCGCATCGGCCT	This study
Bphy_7407_R	GCGCgaattcCCAACGATGTAGCACTTGCT	This study
Bphy_7407_veri_F	CGTCTGTCTGGTTCACCG	This study
Bphy_7407_veri_R	AGGAAGGTTGTAGGGTGCAT	This study
Bphy_7407_c_F_nat_EcoRI	GCGCGAATTCGGCAGCGTCTATCTGAACTA	This study
Bphy_7407_c_R_XbaI	GCGCTCTAGACAAATGGAAAGAGAACTCGCA	This study
Trim_stop_R_NdeI	GCGCCATATGGTAGATATGACGACAGGA	This study
pSHAFT_F	CTTCAGCTGATGTGTGATAACATACT	
Bphy_7808_F	GGCGTGGACTATGTGTCGTA	(Lardi et al. 2017)
Bphy_7808_R	GATGCCCTTCGAGATGTTGT	(Lardi et al. 2017)
rpoD_F	CAATGCTCCGTTGCTGAATA	(Lardi et al. 2020)
rpoD_R	TTCATCTTCGTCATCGTCCA	(Lardi et al. 2020)

Supplementary References

Kovach, M. E., Elzer, P. H., Hill, D. S. et al. Four new derivatives of the broad-host-range cloning vector pBBR1MCS, carrying different antibiotic-resistance cassettes. *Gene* 1995; **166/1**: 175–176.

Lardi, M., Liu, Y., Purtschert, G. et al. Transcriptome Analysis of *Paraburkholderia phymatum* under Nitrogen Starvation and during Symbiosis with *Phaseolus Vulgaris*. *Genes* 2017 **8/12**: 389.

Lardi, M., Liu, Y., Hug, S. et al. *Paraburkholderia phymatum* STM815 σ54 Controls Utilization of Dicarboxylates, Motility, and T6SS-b Expression. *Nitrogen* 2020; **1/2**: 81–98.

Moulin, L., Munive, A., Dreyfus, B. et al. Nodulation of legumes by members of the beta-subclass of *Proteobacteria*. *Nature* 2001; **411/6840**: 948–950.

Figurski and Helsinki. Replication of an origin-containing derivative of plasmid RK2 dependent on a plasmid function provided in trans. *Proceedings of the National Academy of Sciences of the United States of America* 1979; **76**: 1648-1652.

Shastri, S., Spiewak, H. L., Sofoluwe, A. et al. An efficient system for the generation of marked genetic mutants in members of the genus *Burkholderia. Plasmid* 2017; **89**: 49–56.
